# Growing skull hemangioma: first and unique description in a patient with Klippel–Trénaunay–Weber syndrome

**DOI:** 10.1007/s00701-016-3012-0

**Published:** 2016-11-07

**Authors:** Lars E. van der Loo, Jan Beckervordersandforth, Albert J. Colon, Olaf E. M. G. Schijns

**Affiliations:** 1 Department of Neurosurgery, Maastricht University Medical Centre, PO Box 5800, 6202 AZ Maastricht, The Netherlands; 2 Department of Pathology, Maastricht University Medical Centre, Maastricht, The Netherlands; 3 Academic Centre for Epileptology, Kempenhaeghe/Maastricht University Medical Centre, Heeze Maastricht, The Netherlands

**Keywords:** Klippel–Trénaunay–Weber syndrome, Skull hemangioma, Hemangioma, Skull tumor, Surgical pathology

## Abstract

We present the first and unique case of a rapid-growing skull hemangioma in a patient with Klippel–Trénaunay–Weber syndrome. This case report provides evidence that not all rapid-growing, osteolytic skull lesions need to have a malignant character but certainly need a histopathological verification. This material offers insight into the list of rare pathological diagnoses in an infrequent syndrome.

## Introduction

Klippel–Trénaunay–Weber syndrome is a rare, congenital syndrome of unknown etiology, first described in 1900 by the French physicians Maurice Klippel and Paul Trénaunay and in 1907 by the English physician Frederic Parkes Weber. The syndrome is characterized by a triad of port-wine stain, varicose veins, and soft tissue and bony hypertrophy, potentially leading to various types of tumors, including bone and soft tissue tumors [1]. The diagnosis of a skull hemangioma in a patient with Klippel–Trénaunay–Weber (KTW) syndrome has not been described previously. Here, we describe the unique case of a patient with drug-resistant epilepsy, Klippel–Trénaunay–Weber syndrome, and the occurrence of a rapid-growing skull lesion.

## Case report

A 45-year-old Caucasian woman presented in the outpatient clinic with a rapidly growing subcutaneous lesion on the left parietal side of the skull. Since 6 months before presentation, she had noticed an increase of the growth rate of this lesion, which was slightly painful when she would lie on it. Previously, a general surgeon at another hospital took a biopsy of this lesion, but had to stop the procedure soon after incision because of substantial blood loss. The diagnosis could not be confirmed at that time. The patient had been known to have Klippel–Trénaunay–Weber syndrome (KTW) since adolescence, presenting with a nevus flammeus on the right side of the face and varicose veins. In the past, she had deep venous thrombosis, pulmonary embolism, and cellulitis, all consistent with KTW syndrome. A tiny lesion was already noticed 2 years earlier when she underwent a right-sided anterior temporal lobectomy plus amygdalohippocampectomy for drug-resistant temporal lobe epilepsy. After this operation, she remained seizure-free.

**Fig. 1:**
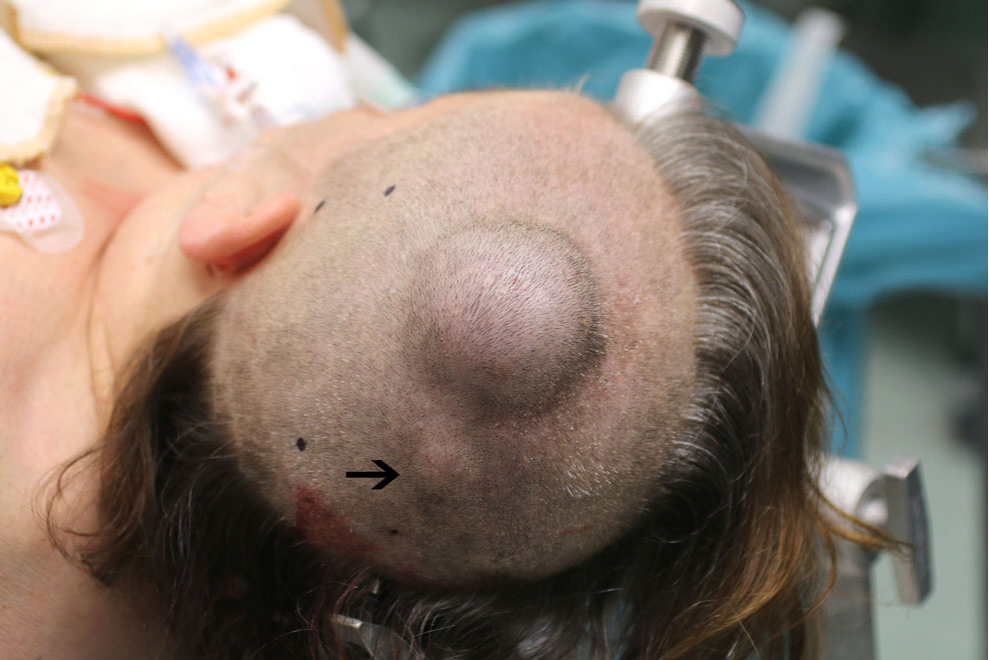
Left parietal subcutaneous tumor, evidently protruding from the skull surface. A second but smaller lesion (*arrow*) is present more posterior and inferior to the large lesion

Clinical examination revealed a left parietal painless subcutaneous tumor of hard consistency, fixated to the skull but not to the skin, approximately 5 cm in diameter and protruding out about 3 cm from the skull surface (Fig. 1). There were no skin defects. Additionally, a smaller but highly similar lesion (diameter 2.2 cm, protruding 0.8 cm) was palpated more inferior, close to the lambdoid suture near the midline.

**Fig. 2:**
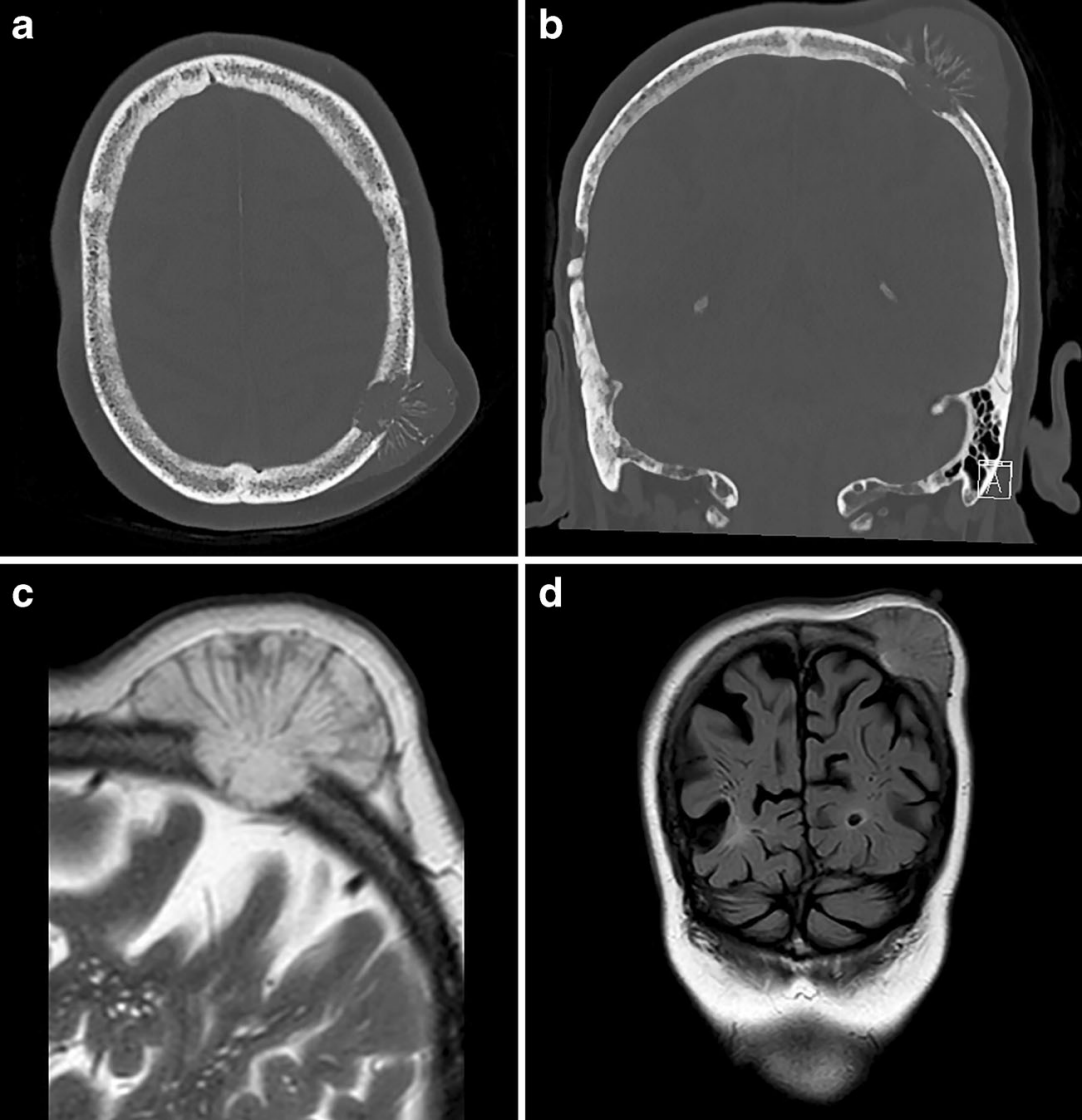
**a** Transverse and **b** coronal CT, windowed to bone setting, demonstrating destruction of both the external and internal tabula of the skull. Extracranial radial striped calcified fragments are present. **c** Sagittal T2-weighted and **d** coronal FLAIR MRI, demonstrating growth to the level of the dura mater, without parenchymal involvement

Computed tomography (CT) of the skull showed an osteolytic lesion with a diameter of 4.5 cm in the left parietal bone with destruction of both the external and internal tabula of the skull. The extracranial portion of the swelling consisted of a radial striped lesion without skin defect (Fig. 2a–b). The second osteolytic lesion had a diameter of 1.4 cm without an extracranial swelling and destruction of only the external tabula of the skull. Contrast-enhanced magnetic resonance imaging (MRI) demonstrated invasion of the tumor to the level of the dura mater, without dural enhancement or parenchymal involvement (Fig. 2c–d). The first radiological differential diagnosis was a metastatic skull lesion from an unknown primary tumor.

**Fig. 3:**
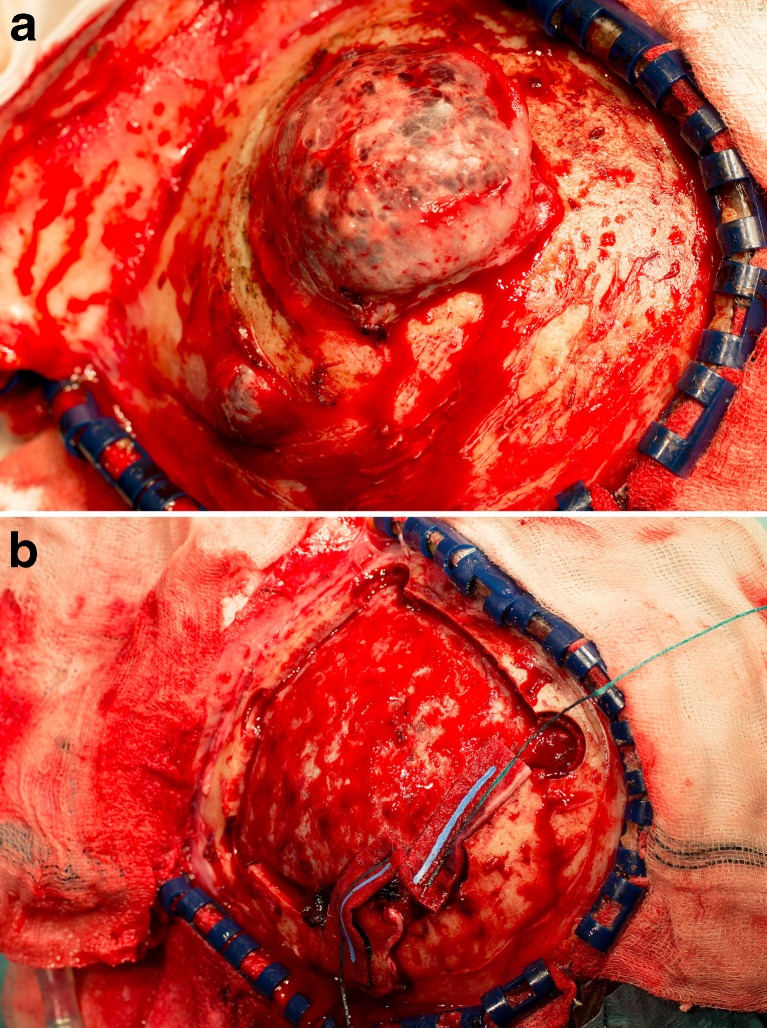
**a** The tumor was fixated to the skull, but not to the skin and subcutaneous tissue, which could easily be separated from the tumor surface. **b** No dural defects were detected after removal of the bone flap

Because of evident growth and a probable malignant tumor, it was decided to perform a left-sided parietal craniectomy with resection of both lesions. The skin and subcutaneous tissue could be easily separated from the lesion (Fig. 3a). After craniectomy, both hard, skull-fixed lesions could be resected. As suspected, there was no dural involvement (Fig. 3b). After craniectomy and meticulous hemostasis, a methyl methacrylate (Palacos®, Zimmer Biomet, Warsaw, IN, USA) plasty was inserted and fixated by mini-plates. The patient had a quick recovery and left the hospital 4 days after operation.

## Histopathology

**Fig. 4:**
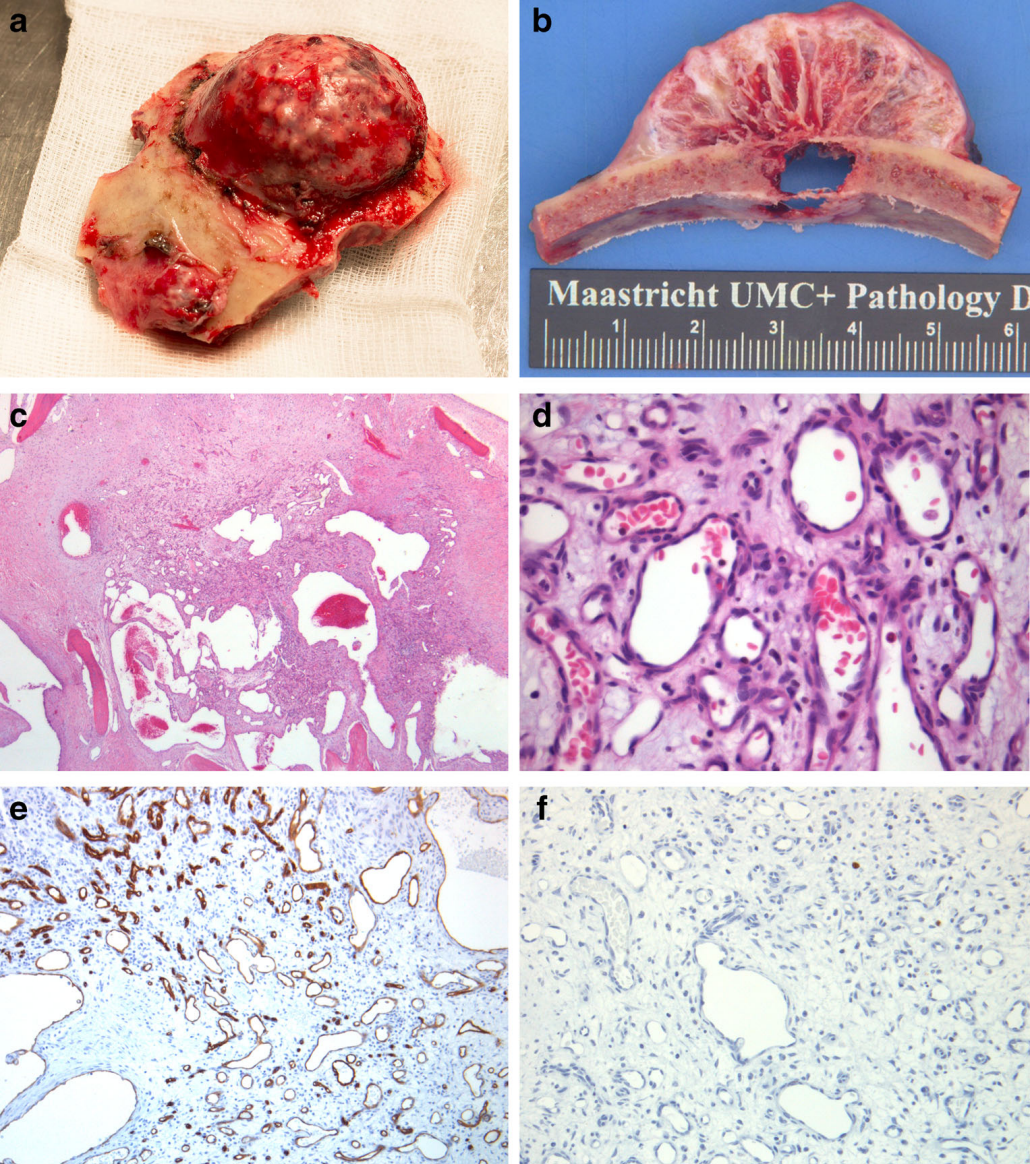
**a** Macroscopic image of both tumors fixated in the bone. **b** Evident infiltration of the tabula interna by the larger tumor.** c**, **d** Hematoxylin and eosin (H & E) stain (magnification ×25 and ×100) showing capillary proliferation. **e** Immunohistochemistry for CD34 (magnification ×100) showed normal capillary endothelial distribution. **f** MIB-1 (magnification ×200) demonstrated no immunoreaction

The resected bone flap showed a hard, bone-like, mushroom-shaped tumor (Fig. 4a). Underneath the large tumor, significant bone erosion was present with destruction of both the tabula interna and externa (Fig. 4b). The second, smaller tumor was similar in appearance with destruction of only the external tabula. Tissue was decalcified in two manners, using Kristensen’s methodology and RDO Rapid Decalcifier. Microscopically, collagenous proliferation with an evident increase in small capillaries was seen (Fig. 4c and d). These vascular structures were lined with normally differentiated endothelium without atypical proliferation. No mitotic spindles were identified. There was no evidence for malignant transformation. There was immunoreaction for anti-smooth muscle antibody (ASMA). Blood vessels showed positive endothelial markers CD31 and CD34 (Fig. 4e). There was no immunoreaction for tumor proliferation marker MIB-1 (Fig. 4f). The histopathological diagnosis of a benign vascular lesion, consistent with hemangioma, was made.

## Discussion

We present, to the best of our knowledge, the first and unique case description of a skull hemangioma in a patient with KTW syndrome.

KTW syndrome is a rare congenital malformation syndrome of unknown etiology. Although the clinical presentation can be extremely variable, the characteristic triad is an association of (cutaneous) capillary malformations, bone, and soft tissue hypertrophy and varicosity [1]. In our case, the patient also had drug-resistant epilepsy, however no association between the KTW syndrome and epilepsy could be identified in the literature.

There are numerous reports about various types of tumors found in patients with KTW syndrome, including malignant peripheral nerve sheath tumor [6], angiosarcoma [3, 6], astrocytoma [2], hemangiopericytoma [4], and meningioma [8]. The fact that heterogeneous tumor types and localizations are still emerging reinforces the fact that this syndrome has a highly variable clinical expression.

In this patient, after a multidisciplinary work-up, the tumor was resected radically and a histopathological diagnosis of hemangioma was confirmed. Hemangiomas are benign tumors and often grow slowly, although as demonstrated by this case, a rapid-growing lesion can also occur and is not necessarily associated with malignancy. Malignant transformation of benign vascular tumors is uncommon and described only in a single case report [7].

Radiological diagnosis in our case included metastasis from an unknown primary tumor. Skull metastases are seen in 15–25 % of all cancer patients and can originate from breast, lung, prostate, thyroid, and renal cell cancer, as well as from hematologic malignancies such as lymphoma, leukemia, and multiple myeloma [5]. It is not uncommon for malignancies to present with solitary skull metastases [5]. The occurrence of two similar tumors very close to each other, as well as rapid growth, supported the differential diagnosis of metastasis, but in the end, radiological analysis did not contribute to the definitive diagnosis of hemangioma.

This case report adds to the literature of highly variable tumor occurrence in KTW syndrome. A hemangioma in the skull has never been described in this syndrome, emphasizing that not only the type of tumor but also the location can be highly variable.

CT, computed tomography; KTW, Klippel–Trénaunay–Weber; MRI, magnetic resonance imaging.
